# AMPA receptor diffusional trapping machinery as an early therapeutic target in neurodegenerative and neuropsychiatric disorders

**DOI:** 10.1186/s40035-025-00470-z

**Published:** 2025-02-11

**Authors:** Daniel Choquet, Patricio Opazo, Hongyu Zhang

**Affiliations:** 1 Univ. Bordeaux, CNRS, Interdisciplinary Institute for Neuroscience, IINS, UMR 5297, 33000 Bordeaux, France; 2 Univ. Bordeaux, CNRS, INSERM, Bordeaux Imaging Center, BIC, UAR 3420, US 4, 33000 Bordeaux, France; 3 UK Dementia Research Institute, Centre for Discovery Brain Sciences, University of Edinburgh, Chancellor’s Building, Edinburgh, EH16 4SB UK; 4 Department of Biomedicine, University of Bergen, 5009 Bergen, Norway; 5 Mohn Research Center for the Brain, University of Bergen, 5009 Bergen, Norway; 6 Department of Radiology, Haukeland University Hospital, 5021 Bergen, Norway

**Keywords:** AMPA receptors, Diffusional trapping, Huntington’s disease, Alzheimer’s disease, Stress, Depression

## Abstract

Over the past two decades, there has been a growing recognition of the physiological importance and pathological implications surrounding the surface diffusion of AMPA receptors (AMPARs) and their diffusional trapping at synapses. AMPAR surface diffusion entails the thermally powered random Brownian lateral movement of these receptors within the plasma membrane, facilitating dynamic exchanges between synaptic and extrasynaptic compartments. This process also enables the activity-dependent diffusional trapping and accumulation of AMPARs at synapses through transient binding to synaptic anchoring slots. Recent research highlights the critical role of synaptic recruitment of AMPARs via diffusional trapping in fundamental neural processes such as the development of the early phases of long-term potentiation (LTP), contextual fear memory, memory consolidation, and sensory input-induced cortical remapping. Furthermore, studies underscore that regulation of AMPAR diffusional trapping is altered across various neurological disease models, including Huntington’s disease (HD), Alzheimer’s disease (AD), and stress-related disorders like depression. Notably, pharmacological interventions aimed at correcting deficits in AMPAR diffusional trapping have demonstrated efficacy in restoring synapse numbers, LTP, and memory functions in these diverse disease models, despite their distinct pathogenic mechanisms. This review provides current insights into the molecular mechanisms underlying the dysregulation of AMPAR diffusional trapping, emphasizing its role as a converging point for multiple pathological signaling pathways. We propose that targeting AMPAR diffusional trapping represents a promising early therapeutic strategy to mitigate synaptic plasticity and memory deficits in a spectrum of brain disorders, encompassing but not limited to HD, AD, and stress-related conditions. This approach underscores an integrated therapeutic target amidst the complexity of these neurodegenerative and neuropsychiatric diseases.

## Background

One major cellular correlate of learning and memory is synaptic plasticity, which refers to the ability of neurons to modify the efficacy of communication at specialized neuronal junctions called synapses in response to specific patterns of activity. These dynamic, bidirectional, and reversible changes in synaptic efficacy allow the storage and utilization of large amounts of information. The vast majority of synapses in the mammalian central nervous system (CNS) are chemical synapses, where information is conveyed through the release of neurotransmitters from the presynaptic terminal and their binding to postsynaptic receptors. Synaptic plasticity can thus arise from changes in the probability of neurotransmitter release (presynaptic mechanisms) or from modifications in the number, composition, biophysical properties (e.g. conductance or open probability), and nanoscale positioning of neurotransmitter receptors relative to presynaptic release sites. These latter changes determine the sensitivity of postsynaptic neurons to neurotransmitters (postsynaptic mechanisms) [1].

At excitatory synapses, glutamate, the primary excitatory neurotransmitter in the CNS, activates five main classes of glutamate receptors: four ionotropic receptors (AMPA receptors [AMPARs], NMDA receptors [NMDARs], kainate receptors, and delta receptors [GluD]) [2], and one class of metabotropic receptors (G-protein-coupled metabotropic glutamate receptors [mGluRs]). All these classes of glutamate receptors are critically involved in the induction, expression, and/or modulation of synaptic plasticity. The most extensively studied forms of long-lasting synaptic plasticity in the vertebrate CNS are NMDAR-dependent long-term potentiation (LTP) and long-term depression (LTD) of synaptic transmission. While presynaptic mechanisms may be involved under certain conditions, NMDAR-dependent LTP and LTD are triggered by the activation of postsynaptic NMDARs and are expressed through an increase and decrease in the synaptic abundance of AMPAR complexes, and/or changes in their subunit composition [1]. Consequently, tremendous effort has been devoted to understanding the dynamic regulation of AMPAR trafficking into and out of synapses.

AMPAR trafficking involves several key processes: intracellular transport, endocytosis/exocytosis, endosomal recycling, surface diffusion, diffusional trapping at synapses, and degradation. Newly synthesized receptors are transported intracellularly in vesicles along microtubules within dendrites, where they can be delivered by exocytosis next to synapses [3, 4]. However, AMPARs at synapses were initially thought to be stable until the late 1990s, when it was discovered that they constantly undergo exocytosis and endocytosis, allowing them to recycle continuously between the neuronal surface and intracellular pools [5–9]. This understanding was further expanded in the early 2000s, with the discovery that AMPARs diffuse rapidly within the plasma membrane via thermally powered Brownian diffusion, enabling their exchange between synaptic and extrasynaptic compartments and their reversible trapping at synapses through binding to various intracellular and extracellular scaffolds [10–12]. Together, these trafficking processes establish a dynamic equilibrium among intracellular, synaptic, and extrasynaptic compartments, which ultimately determines the number of AMPARs at synapses. While intracellular trafficking facilitates coarse-scale receptor distribution along dendrites, local regulation within or near the synapse through endocytosis/exocytosis, surface diffusion, and diffusional trapping allows fine-tuned control of the number of AMPARs at synapses.

Surface diffusion has received increasing attention due to its unique properties. Firstly, unlike intracellular transport and exocytosis/endocytosis that require energy from the hydrolysis of adenosine triphosphate [13, 14], surface diffusion is driven solely by thermal agitation, resulting in ‘free’ movement that incurs no energetic cost to the cell [1]. Secondly, surface diffusion is highly efficient for short-range displacement within the plasma membrane. For instance, synaptic AMPARs can escape from a 200-nm-diameter region via diffusion within tens of milliseconds [15, 16], and their redistribution between synaptic and extrasynaptic sites occurs within subseconds to tens of seconds [17–21].

Thirdly, in contrast to AMPAR endocytosis and exocytosis, which primarily occur at perisynaptic and extrasynaptic sites [19, 22–28], surface diffusion allows AMPARs to directly reach and leave the postsynaptic density (PSD) as well as to diffuse within the PSD between subdomains [29, 30]. Consequently, surface diffusion plays a pivotal role in synaptic delivery and removal of AMPARs: exocytosed AMPARs at perisynaptic/extrasynaptic sites require surface diffusion to reach the PSD [17, 19]; while endocytosis may require AMPARs to diffuse from the PSD to endocytic zones located at perisynaptic/extrasynaptic sites.

Fourthly, surface diffusion is driven by weak thermal forces that are easily influenced by protein–protein interactions, which are often transient and reversible, between AMPARs and synaptic trapping slots at the PSD. This dynamic interaction allows synaptic AMPARs to constantly switch between a mobile (diffusional) state and an immobile (trapped) state in a process known as reversible diffusional trapping at synapses [1, 4, 11]. The strength of diffusional trapping can be finely modulated during synaptic plasticity through changes in the number, affinity, availability, and localization of synaptic trapping slots, leading to alterations in the abundance and nanoscale positioning of synaptic AMPARs, which ultimately determine the efficiency of synaptic transmission [29–37]. Thus, the interplay between surface diffusion and diffusional trapping at synapses provides an efficient, precise and energy-saving mechanism underlying synaptic plasticity.

Recent studies have demonstrated that AMPAR surface diffusion plays a critical role in LTP. Using AMPAR immobilization approaches in-vitro and in-vivo, these studies showed that the initial phase of LTP (the first few minutes) is mediated by the rapid recruitment of diffusive AMPARs already present on the neuronal surface [17, 38]. While subsequent exocytosis of AMPARs is essential for the maintenance of LTP [5, 17, 39, 40], interfering with the diffusion of newly exocytosed AMPARs abolishes LTP maintenance [17]. This suggests that newly exocytosed AMPARs at extrasynaptic sites must diffuse to synaptic sites to sustain LTP. Moreover, blocking AMPAR surface diffusion in the hippocampus in-vivo using various crosslinking approaches significantly impairs hippocampus-dependent contextual fear memory [17, 38]. Similar immobilization paradigms have shown that AMPAR surface diffusion is also crucial for in vivo memory consolidation [41] and LTP in the barrel cortex induced by whisker stimulation [42]. Collectively, these findings suggest that the synaptic delivery of AMPARs via surface diffusion is an essential mechanism for the expression of LTP, various forms of memory, cortical remapping, and adaptive behaviors during sensory experiences [42, 43].

**Fig. 1:**
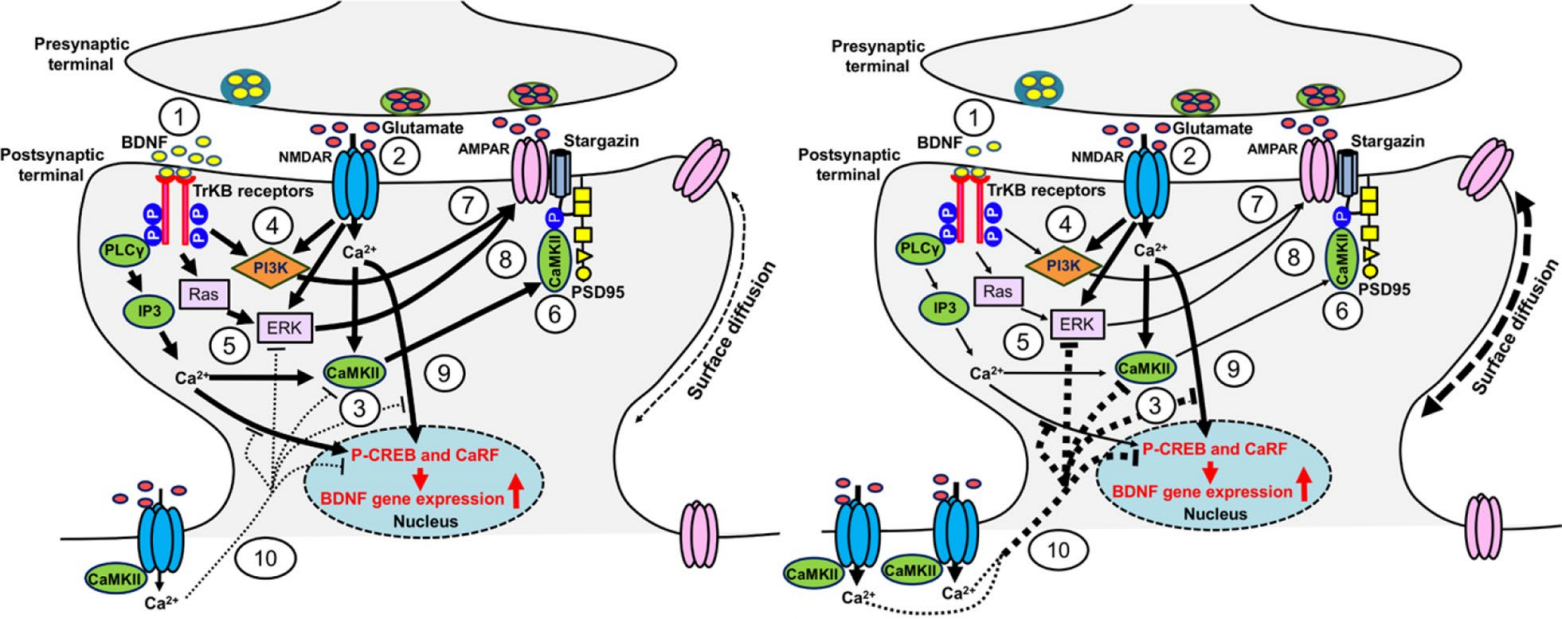
Effects of BDNF-TrkB and NMDAR signaling on AMPAR diffusional trapping under physiological and pathological conditions. Left panel (physiological condition): BDNF binding to TrKB receptors (**1**) and Ca^2+^ influx via synaptic NMDARs (**2**) activate multiple signaling cascades, including CaMKII (**3**), PI3K (**4**), and Ras-ERK (**5**). Activated CaMKII translocates to the postsynaptic density (PSD), where it phosphorylates stargazin (TARP γ2), potentiates the interaction between PSD-95 and stargazin, and stabilizes AMPAR anchoring (**6**), thereby decreasing AMPAR surface diffusion. PI3K and Ras-ERK also contribute to the stabilization of synaptic AMPARs, although the direct trapping mechanism remains to be explored (**7**, **8**). Elevated Ca^2+^ levels promote *BDNF* gene expression by facilitating the binding of transcription factor, such as CREB and CaRF, to regulatory elements of *BDNF* (**9**). Extrasynaptic GluN2B-NMDARs counteract the effects of synaptic NMDARs by triggering CREB and ERK shut-off and preventing CaMKII translocation to synapses (**10**). Right panel (pathological conditions, e.g. HD and AD): impaired BDNF-TrkB signaling leads to deficiencies in CaMKII, PI3K, and Ras-ERK pathways. Additionally, abnormally enhanced extrasynaptic NMDAR signaling significantly promotes a dominant CREB shut-off pathway, blocking the induction of *BDNF* expression (**10**). It also shuts off ERK and sequesters active CaMKII due to CaMKII's high binding affinity for GluN2B-containing NMDARs, preventing CaMKII from translocating to synapses. The combined effects of impaired BDNF-TrKB signaling and imbalanced synaptic and extrasynaptic NMDAR signaling synergistically weaken the diffusion trapping mechanism, destabilize synaptic AMPARs, and increase AMPAR surface mobility

Mechanistically, we have demonstrated that during LTP, AMPAR complexes are recruited to synapses through the diffusional trapping of transmembrane AMPA receptor regulatory proteins (TARPs), such as Stargazin (TARP γ2), by the major postsynaptic density scaffold protein, PSD-95 [4, 11, 31, 44]. Additionally, the involvement of other TARP family members, such as γ8, in this diffusion-trapping process has also been proposed [45–47]. Our findings, along with those of others, indicate that NMDAR activation leads to the activation of calcium/calmodulin-dependent protein kinase II (CaMKII), resulting in the phosphorylation of Stargazin and γ8. This phosphorylation enhances their binding to the synaptic scaffold PSD-95 (Fig. 1a) [31, 44, 48]. Stargazin forms highly concentrated and dynamic condensates with PSD-95 or PSD-95-assembled postsynaptic complexes through phase separation, reminiscent of Stargazin/PSD-95-mediated AMPAR synaptic clustering and trapping [32]. Importantly, the TARP/PSD-95 interaction is necessary for synaptic AMPAR trafficking and LTP [49]. Recent studies, however, have challenged the traditional view of CaMKII as primarily a kinase in LTP, highlighting instead its critical structural role in synaptic plasticity [50–52]. These findings prompt a reevaluation of the exact mechanisms underlying the activity-dependent diffusional trapping of AMPARs during LTP.

Beyond the critical role of TARP binding to PSD-95, other AMPAR auxiliary subunits and PDZ-domain-containing adhesion proteins—such as members of the Shisa family and neuroligin—likely contribute to activity-dependent AMPAR trapping during LTP and memory [43, 53–55]. There is also growing interest in the role of the extracellular N-terminal domains (NTDs) of AMPARs in stabilizing AMPARs at synapses. These NTDs may interact with extracellular proteins such as pentraxins, cadherins, and noelin, potentially functioning as diffusion-trapping scaffolds. However, the mechanisms by which neuronal activity regulates these interactions remain unclear [45, 56–59]. Notably, recent studies revealing a pH-dependent splaying of certain GluA subunit NTDs could shed new light on how AMPAR NTDs interact with matrix proteins [60–62]. In summary, these findings highlight the complex interplay of molecular mechanisms underlying activity-dependent AMPAR diffusional trapping during LTP, involving TARPs, auxiliary subunits, adhesion proteins, and extracellular interactions. Future research will be essential to unravel how these processes are dynamically regulated to support synaptic plasticity and memory formation.

The pathophysiological role of AMPAR diffusional trapping has been recently demonstrated in several rodent models of neurodegenerative and neuropsychiatric disorders, including Huntington’s disease (HD), Alzheimer’s disease (AD), and depression. Despite their distinct etiologies and pathogenesis, models of HD, AD, and depression exhibit similar dysregulation of AMPAR diffusional trapping, early-stage impairment of hippocampal LTP prior to neuronal loss, and early-onset memory deficits [18, 63, 64]. Notably, pharmacological interventions that re-establish AMPAR diffusional trapping also lead to restoration of synapse numbers, hippocampal LTP, and memory in these models. These findings suggest that dysregulated AMPAR diffusional trapping may serve as a common pathological substrate across these disorders and presents a promising therapeutic target.

In this review, we underscore the notion that dysregulation of AMPAR diffusional trapping may be a unifying and convergent mechanism underlying brain disorders associated with impaired learning and memory. We propose that targeting AMPAR diffusional trapping represents a promising early therapeutic strategy to restore synaptic plasticity and ameliorate memory deficits across a spectrum of neurodegenerative and neuropsychiatric disorders.

## Dysregulation of AMPAR diffusional trapping in HD

HD is an autosomal-dominant neurodegenerative disorder caused by a mutation in the huntingtin gene, characterized by more than 36 CAG repeats. This mutation results in the production of a mutant huntingtin protein with expanded polyglutamine repeats (polyQ) [65]. Clinically, HD manifests as motor dysfunction, psychiatric disturbance (eg. depression), and cognitive deficits. These symptoms have traditionally been attributed to the degeneration or loss of medium-sized spiny neurons in the striatum and cortical neurons. However, increasing evidence indicates that HD murine and primate models, as well as early-stage HD patients and clinically asymptomatic HD mutation carriers, exhibit cognitive deficits and psychiatric disturbance well before the appearance of classical neuropathology or motor symptoms [66–73]. This suggests that cellular dysfunction, rather than neuronal loss, underlies the initial development of the disease.

### Early deficits in synaptic plasticity prior to neurodegeneration in different HD mouse models

Various HD mouse models exhibit impaired hippocampal synaptic plasticity at pre- or early-symptomatic stage. For example, R6/1 heterozygous transgenic mouse model, one of the most widely used HD models, which overexpresses the first exon of human huntingtin with approximately 115 polyQ repeats, show deficits in hippocampal LTP at 5 weeks of age [74], well before the onset of motor deficits and striatal neuron loss observed at 12 weeks of age [75, 76]. Similarly, R6/2 heterozygous transgenic mouse model, which overexpresses the first exon of human huntingtin with 150 polyQ repeats, show reduced hippocampal LTP from as early as 5 weeks, coinciding with the onset of motor symptoms between 5 and 18 weeks [69, 77–79]. In addition to LTP deficits, R6/2 mice display abnormally enhanced hippocampal LTD between 5 and 18 weeks of age [79], while R6/1 mice regain the ability to support LTD, which is typically lost in the CA1 region of the hippocampus, at 12 weeks of age [80].

Although these transgenic mice exhibit a rapid onset of phenotypes advantageous for mechanistic studies, overexpression of the protein may induce undesired artifacts and the lack of full-length huntingtin protein may not perfectly replicate the human condition. A complementary HD model, the knock-in (KI) mouse model, incorporates the mutation directly into its huntingtin gene, closely replicating the genetic aspects of the human condition. This model expresses the huntingtin protein at endogenous levels and, similar to HD patients, exhibits a delayed onset of overt symptoms [81]. The Hdh^Q92^ and Hdh^Q111^ knock-in mice, which have 92 or 111 CAG repeats inserted into the huntingtin gene, show a significant reduction in hippocampal LTP around 8–10 weeks of age, months before the onset of motor symptoms [64, 82]. Similarly, Hdh^CAG140/+^ knock-in mouse model, which has 140 polyQ repeats inserted into the mouse huntingtin protein, exhibit hippocampal LTP deficits at 8 weeks of age [83]. Another complementary mouse model is the yeast artificial chromosome (YAC) transgenic mice, which express full-length huntingtin. In YAC72 mice, which carry 72 polyQ repeats, high-frequency stimulation (HFS) failed to induce hippocampal LTP at 10 months of age, preceding selective striatal neurodegeneration first observed at 12 months [84]. Moreover, HFS protocols induced depression rather than potentiation in 10-month-old YAC72 mice [84].

Deficits in synaptic plasticity appear to arise mainly from postsynaptic mechanisms, as studies in Hdh^Q111/Q111^ and HdhCAG^140/+^ knock-in models as well as in R6/1 and R6/2 heterozygous transgenic mouse models found no detectable differences in presynaptic parameters in the CA1 region of the hippocampus [79, 80, 82, 83].

In summary, these findings suggest that HD begins with disturbance of synaptic plasticity, which subsequently progresses to motor pathology and neurodegeneration.

### Dysregulation of AMPAR diffusional trapping via abnormal brain-derived neurotrophic factor (BDNF) signalling pathways

We recently identified dysregulation of AMPAR diffusional trapping as one key postsynaptic mechanism underlying LTP deficits in HD. The HD-causing mutation leads to the destabilization of AMPARs at the postsynaptic membrane and increases the speed of AMPAR surface diffusion under basal condition or after chemically-induced LTP in various complementary HD models. These models include ectopic expression of either wild-type or polyQ–exon1, as well as polyQ-full-length huntingtin constructs in rat primary hippocampal neurons, hippocampal neurons from R6/1 and R6/2 heterozygous transgenic mice, and homozygous Hdh^Q111/Q111^ and heterozygous Hdh^CAG140/+^ knock-in mouse models [64].

The upstream signaling pathways that impair the diffusional trapping machinery in HD appear to involve BDNF and its high-affinity receptor, tropomyosin receptor kinase B (TrkB) [64, 85–90]. Activation of BDNF–TrkB can trigger three key signaling cascades that impact synaptic recruitment of AMPARs: PLCγ (phospholipase Cγ)-CaMKII, phosphatidylinositol 3-kinase (PI3K)-Akt, and Ras-Raf-MEK-extracellular-signal-regulated kinase (ERK) signaling pathways (Fig. 1) [91–94].

Critically, these signaling pathways are major regulators of diffusional trapping of AMPARs. First, synaptic CaMKII is essential for immobilizing AMPARs at synapses under basal conditions and after LTP via phosphorylation of TARPs, facilitating their binding to the synaptic scaffolding protein PSD-95 [44, 95]. Second, the PI3K signaling pathway is involved in immobilizing surface AMPARs, as inhibiting PI3K with LY294002 increases the surface mobility of recombinant AMPARs in hippocampal neurons [96]. Importantly, BDNF induces the translocation of PSD-95, a synaptic scaffold critical for AMPAR immobilization, to dendrites through PI3K–AKT signalling [97]. Last, while the role of ERK signaling in AMPAR diffusion is not well understood, the ERK pathway has been shown to increase AMPAR exocytosis, thereby expanding the extrasynaptic pool of mobile AMPAR available for synaptic anchoring [26].

Various HD mouse models, as well as HD subjects, exhibit reductions of cortical and hippocampal BDNF mRNA and protein [64, 85, 88–90, 98] as well as BDNF intracellular transport [64, 88, 98, 99]. The downstream signal transduction of BDNF–TrkB is also reduced in various HD mouse models [86, 87, 100]. Impaired activation of CaMKII [64, 85], PI3K [87], and ERK signaling pathways [101, 102] has been reported in different HD knock-in mouse models. Additionally, the interaction between PSD-95 and stargazin (TARP γ-2), a key component of diffusional trapping machinery [20, 44], is dramatically disrupted in the hippocampus of Hdh^Q111/Q111^ knock-in HD mouse models and in hippocampal neurons overexpressing full-length huntingtin, despite the unchanged protein levels of stargazin and PSD-95 [64]. This hypothesis is further corroborated by evidence that exogenous BDNF application rescues the diffusional trapping of AMPARs [64] and restores HD-associated synaptic plasticity [82]. This rescue effect on AMPAR surface diffusion is blocked by the BDNF scavenger TrkB-Fc, the CaMKII inhibitor KN-93, or in neurons expressing ΔC stargazin (ΔC Stg), in which the binding domain to PDZ-domain scaffold proteins like PSD-95 is deleted [64].

### Potential role of extrasynaptic NMDARs in AMPAR diffusional trapping deficits in HD

An imbalance between synaptic NMDARs (predominantly composed of GluN2A subunits in mature neurons) and extrasynaptic NMDARs (mainly associated with GluN2B subunits in mature neurons) may also contribute to deficits in AMPAR diffusional trapping [103–105].

Synaptic and extrasynaptic NMDARs exert opposing effects on cyclic AMP (cAMP) response element (CRE)-binding protein (CREB) function and BDNF gene regulation [106]. Calcium entry through synaptic NMDAR induces CREB activity and BDNF gene transcription, whereas calcium entry through extrasynaptic NMDARs activates a dominant CREB shut-off pathway that blocks BDNF gene expression [107]. Consequently, overactivation of extrasynaptic NMDARs may lead to a decrease in BDNF expression, critically impacting the diffusional trapping of AMPARs. Alternatively, extrasynaptic GluN2B-NMDARs in HD models may destabilize synaptic AMPAR by sequestering CaMKII at extrasynaptic sites, preventing it from translocating to synapses to immobilize AMPARs due to the higher binding affinity of GluN2B for CaMKII compared to synaptic GluN2A-containing NMDARs [108].

Taken together, these studies suggest that an imbalance between synaptic and extrasynaptic NMDAR activity can impair BDNF production and TrkB signaling, ultimately leading to the synaptic destabilization of AMPARs. This sequence of events may create a vicious cycle, where reduced BDNF expression exacerbates NMDAR imbalances and AMPAR instability, further amplifying synaptic dysfunction. Over time, this dysregulation contributes to the progressive breakdown of neuronal networks, resulting in the behavioral deficits characteristic of HD. Interventions targeting this cellular axis—such as enhancing BDNF signaling, restoring NMDAR balance, or stabilizing AMPARs—offer potential strategies to disrupt this cycle and mitigate HD-related synaptic and behavioral impairments.

## Dysregulation of AMPAR diffusional trapping in AD

AD is a neurodegenerative disease clinically characterized by progressive cognitive impairment, with early-onset symptoms including memory deficits and neuropsychiatric manifestations such as depression. Traditionally, these symptoms were thought to result primarily from neuronal degeneration. However, substantial evidence suggests that AD begins with synaptic failure prior to overt neuronal degeneration [109]. The clinical relevance of the “synaptotoxic” hypothesis is underscored by several studies indicating that synaptic loss correlates better with the severity of cognitive deficits in AD than neuronal loss [110, 111]. The exact causes of AD remain unclear, but amyloid-β (Aβ) and tau proteins are believed to play a central role in its etiology and pathogenesis.

### Early deficits in synaptic plasticity prior to neurodegeneration in different AD mouse models

It is now well accepted that soluble and oligomeric forms of Aβ (oAβ) weaken synaptic transmission by both preventing LTP [112, 113] and facilitating LTD [114, 115]. Regarding LTP, numerous studies have shown that both synthetic and naturally secreted forms of oAβ consistently inhibit the induction of LTP in vitro and in vivo [112, 113]. Consistent with these findings, LTP is impaired in transgenic AD mouse models even before the accumulation of amyloid plaques. Regarding LTD, several studies have shown that oAβ not only facilitates the induction of LTD [114, 115], but that chronic application of oAβ is sufficient to weaken synaptic transmission in a manner resembling LTD.

Because both LTP and LTD rely heavily on NMDAR activation, calcium-dependent signaling and AMPAR trafficking, oAβ may interfere with one or all of these cellular processes [116–118]. On the neuronal surface, oAβ can disrupt NMDAR function either directly, through interaction [119, 120], or indirectly, by interacting with other transmembrane proteins [121, 122]. The critical role of NMDARs in oAβ synaptotoxicity is highlighted by multiple studies demonstrating that NMDAR antagonists fully rescued the detrimental effects of oAβ [114, 123–125]. GluN2B-containing NMDARs are particularly associated with synaptotoxicity as the specific antagonist ifenprodil rescued both the LTP deficits and synaptic loss mediated by oAβ [126–129].

Downstream of NMDAR activation, it is thought that calcium shifts the balance between CaMKII and the calcium-dependent phosphatase calcineurin at synapses toward phosphatase activation, which eventually results in AMPAR dephosphorylation, AMPAR endocytosis and synaptic weakening [116, 130, 131]. The critical role of AMPAR endocytosis in oAβ-mediated synaptotoxicity is highlighted by studies showing that blocking AMPAR endocytosis [114] or the signalling associated with AMPAR internalization [132] prevented synaptotoxicity. Once internalized, AMPARs undergo ubiquitination and are sorted into late endosomes for degradation, resulting in a decreased number on the neuronal surface [133].

### Dysregulation of AMPAR diffusional trapping in AD via abnormal CaMKII signalling

In addition to facilitating endocytosis, it is possible that oAβ may impact the surface diffusion of AMPARs. Because the endocytic machinery is located at peri- and extrasynaptic sites [27], AMPAR destabilization and lateral diffusion to these sites are likely prerequisites for the endocytosis of synaptic AMPARs [118]. Our previous work showed that the diffusional trapping and stabilization of AMPAR at synapses strongly rely on CaMKII-dependent phosphorylation of stargazin [31, 44]. This raises the possibility that the oAβ-mediated shift toward phosphatase activity might trigger the destabilization and escape of synaptic AMPARs. Consistent with this hypothesis, we demonstrated that overexpression of the amyloid-precursor protein (APP) or prolonged exposure to oAβ is sufficient to destabilize synaptic AMPARs [63]. Importantly, we found that AMPAR destabilization contributes to oAβ-mediated synaptotoxicity, as preventing it via a crosslinking approach fully rescued synaptic loss [63]. Mechanistically, we showed that oAβ mediates the destabilization of surface AMPAR via activation of GluN2B-containing NMDARs and, surprisingly, through the activation of CaMKII. We further confirmed that CaMKII is required for synaptotoxicity as KN-93 and tatCN21, two mechanistically distinct CaMKII inhibitors, completely rescued the oAβ-mediated deficits in LTP and synaptic loss [63].

### Potential role of extrasynaptic NMDARs in AMPAR diffusional trapping deficits in AD

While the oAβ-mediated activation of CaMKII aligns with the well-documented structural and functional coupling between GluN2B and CaMKII [134, 135], as well as the contributing role of CaMKII activity in oAβ toxicity [136–139], it is at odds with our previous studies showing that NMDAR and CaMKII activation during LTP is necessary for synaptic stabilization of AMPAR [31, 44]. We propose that these discrepancies result from the distinct subcellular localizations of NMDARs and CaMKII engaged during LTP compared to those activated in response to oAβ exposure. While LTP relies on the exclusive activation of synaptic NMDAR and the translocation of CaMKII to activated synapses [44, 135, 140], extracellular oAβ triggers prominent activation of extrasynaptic NMDAR and therefore non-synaptic CaMKII [63]. In fact, several studies have shown that selective activation of extrasynaptic GluN2B-containing NMDARs is responsible for the oAβ-mediated LTP impairments [141–143].

Because extrasynaptic NMDAR signaling not only antagonizes but also dominates over synaptic signaling [103, 107], exposure to oAβ prevents subsequent activity-dependent synaptic translocation of CaMKII and the diffusional trapping of AMPAR during LTP [63, 144–146]. Prolonged exposure to oAβ likely leads to a reduction in the synaptic pool of active CaMKII, disruption of the stargazin–PSD-95 interaction, destabilization and escape of synaptic AMPARs, and ultimately, synaptic loss. This hypothesis is consistent with findings of reduced synaptic pool of active CaMKII in transgenic animal models of AD and the observation that restoring the synaptic CaMKII levels normalizes synaptic function [144]. The clinical relevance of these findings is further supported by a study in postmortem AD brains that showed a significant redistribution of active CaMKII from the synapse to the cytoplasm [147]. Remarkably, the relative distribution of active CaMKII strongly predicts the premortem cognitive deficits in these subjects [147]. The critical role of non-synaptic CaMKII activation is further highlighted by studies showing that oAβ leads to the activation and increased association of CaMKII with metabotropic glutamate receptor 5 (mGluR5), which are predominantly localized extrasynaptically [148–150].

Altogether, these findings suggest that prolonged exposure to oAβ leads to a reduction in the synaptic pool of active CaMKII, ultimately resulting in synaptic destabilization of AMPARs. Thus, normalizing synaptic CaMKII levels may be a sensitive strategy for restoring synaptic function during the early synaptotoxic stages of AD. Since the anchoring of CaMKII at synapses strongly relies on its autonomous activation via autophosphorylation at Thr 286 [134, 135], manipulations aimed at facilitating autophosphorylation might prevent CaMKII mislocalization and restore synaptic function in AD models. Consistent with this idea, behavioral training ameliorated learning and memory deficits in the Tg2576 transgenic and in sporadic AD models by increasing CaMKII autophosphorylation [151, 152].

In addition to NMDAR being a putative receptor for oAβ [119, 121], it is well accepted that oAβ can bind to several other postsynaptic receptors, including mGluR5, the prion protein receptor PrP(C) [148, 153], voltage-dependent calcium channels [154], the α7-nicotinic receptor [155], and the ephrin-type B2 receptors [122]. Although these putative oAβ receptors are diverse in nature, they all regulate intracellular Ca^2+^ levels, either directly or indirectly, and consequently influence CaMKII activation. Extensive research showing that disruptions in Ca^2+^ homeostasis are central to AD pathogenesis [156–158] suggests that CaMKII dysfunction and impaired stabilization of synaptic AMPARs might represent key outcomes of Ca^2+^ dysregulation [139].

Notably, there are striking parallels between oAβ and mutant Huntingtin (mHTT) synaptotoxicity. Similar to oAβ, mHTT toxicity is associated with the overactivation of extrasynaptic GluN2B-containing NMDARs (Fig. 1) [103–105] and, as our studies show, with prominent destabilization of synaptic AMPARs [63, 64]. Intriguingly, BDNF levels are also reduced in the brains of AD patients [98], and BDNF has proven to be beneficial both in AD [158] and HD models [64], likely by attenuating extrasynaptic NMDAR signaling [93] and promoting CaMKII autophosphorylation [158]. BDNF might also promote the activation of the PI3K cascade, which, like CaMKII, is critical for the surface stabilization of AMPARs [96] and whose downregulation has been implicated in oAβ-mediated synaptotoxicity [159]. Taken together, these mechanistic similarities are consistent with the notion that dysregulation in AMPAR diffusional trapping may represent a shared mechanism leading to cognitive deficits in these pathologies.

## Dysregulation of AMPAR diffusional trapping in rodent models of stress and depression

Depression (major depressive disorder) is a complex, heterogeneous mood disorder characterized by a persistent feeling of sadness and loss of interest. Etiologically, depression is often linked to stress, with around 80% of depressive episodes preceded by major life events or ongoing stressor in community samples [160–164]. Similar to HD and AD, cognitive impairments, including deficiencies in long-term and working memory, are essential aspects of depression symptomatology [165–167].

The molecular mechanisms underlying depression are not fully understood. Traditionally, the monoamine hypothesis attributes depression to a depletion of monoamines**–**serotonin, norepinephrine, and dopamine—in the CNS. This hypothesis is primarily based on the fact that traditional antidepressants—such as selective serotonin reuptake inhibitors (SSRIs), norepinephrine reuptake inhibitors, tricyclic antidepressants (TCAs), and monoamine oxidase inhibitors—act by raising extracellular monoamine levels. However, monoamine hypothesis has been increasingly challenged over the last two decades [167, 168].

First, conventional monoaminergic drugs display a therapeutic time lag of several weeks to months and show limited efficacy [168, 169]. Second, the selective serotonin reuptake enhancer tianeptine, which decreases extracellular levels of serotonin and primarily targets glutamatergic system, has been proven effective for treating depression [170, 171]. Third, drugs that target glutamate receptors, such as NMDAR antagonists, AMPAR positive allosteric modulators, metabotropic glutamate receptor (mGluR) negative allosteric modulators, also exhibit antidepressant effects in animal models or depressed patients [168, 172–174]. These findings highlight the significant role of the glutamatergic system in the development of depression and the effectiveness of antidepressants.

### Deficits in synaptic plasticity in different mouse models of stress and depression

In rodent models of depression, stress hormones and behavioral stress induce changes in AMPAR trafficking and synaptic plasticity, depending on the type of stressor, timing, and brain regions [167]. Generally, acute stress has bidirectional effects on synaptic plasticity, either enhancing or impairing LTP, whereas chronic stress tends to impair LTP and facilitate LTD in the hippocampus and prefrontal cortex (PFC)**–**two critical regions for learning and memory [175]. Notably, various classes of antidepressants can reverse these changes, and their antidepressant effects are blocked by AMPAR antagonists, suggesting that AMPARs play a central role in mediating the antidepressant effects of these drugs [167, 168, 176].

Corticosterone, a principal glucocorticoid secreted in response to stress, induces biphasic changes in hippocampal synaptic plasticity. Short-term (10 min) application of corticosterone (100 nmol/L) facilitates LTP in hippocampal CA1 pyramidal neurons [177, 178]. Over a few hours, corticosterone increases AMPAR miniature and evoked excitatory postsynaptic current (EPSC) amplitudes, as well as synaptic GluA2-AMPAR content, through intracellular glucocorticoid receptors, potentially occluding further LTP induction [178, 179]. Similarly, in the PFC, a short-term application of corticosterone (100 nmol/L, 20 min) to PFC neurons significantly increases synaptic GluA1-AMPAR content and AMPAR-EPSC amplitude [180]. In contrast, prolonged corticosterone administration reduces the protein levels of GluA2/3-containing AMPARs [181].

Behavioral stressors, such as acute inescapable stress (AIS) and acute restraint plus tailshock stress (ARTS), impair LTP in the hippocampal CA1 and DG regions [18, 182–187] and in the PFC [188–191]. Conversely, acute forced swim stress (AFSS) potentiates LTP in the ventral hippocampus [192] and AMPAR EPSCs in PFC pyramidal neurons via glucocorticoid receptor activation, and enhances PFC-dependent working memory [180]. Moreover, ARTS promotes LTD in the CA1 region of the hippocampus [187, 193] and AFSS facilitates LTD in the dorsal hippocampus [192]. In contrast, chronic stress models such as chronic unpredictable mild stress (CUMS), chronic restraint stress, chronic inescapable shock, and chronic psychosocial stress impair LTP in hippocampal DG [194–196], CA3-CA1 [194, 197–199], and temporoammonic (TA)-CA1 regions [200], and reduce the total or surface levels of AMPARs [201, 202], the total PSD-95 protein levels [201, 203], and LTP in the PFC [189, 204–206]. Furthermore, chronic mild naturalistic stress and CUMS facilitate LTD in the CA1 region of the hippocampus [207, 208].

Taken together, these findings strongly suggest that deficits in synaptic plasticity are a consistent feature of models of stress and depression, likely mediated by impairments in AMPAR trafficking.

### Dysregulation of AMPAR trafficking in rodent models of stress and depression

Consistent with the idea that deficits in AMPAR trafficking might underlie deficits in synaptic plasticity and cognition in stress and depression models, numerous studies have shown that various mechanistically different antidepressants impact AMPAR trafficking and function.

#### Conventional antidepressants

In non-stressed rats, chronic treatment with SSRI paroxetine and the TCA desipramine produces a time- and dose-dependent increase in AMPARs in the membrane without affecting total protein levels [209], suggesting a drug-induced redistribution of AMPARs via trafficking. Similarly, chronic treatment with SSRI fluoxetine upregulates BDNF protein levels in the hippocampus and cortex [210], increases the GluA2 subunit of AMPARs in the synaptic membrane of the retrosplenial granular b cortex [211], and elevates the GluA2 and GluA4 subunits in PFC [212]. Additionally, it increases S845-GluA1, another critical phosphorylation event for the synaptic trafficking of AMPARs, in the hippocampus, PFC and striatum [213].

In line with these findings, the norepinephrine reuptake inhibitor reboxetine elevates BDNF protein levels in the hippocampus and cortex [210] and increases GluA1 and GluA3 protein levels in the PFC [212]. The tetracyclic antidepressant maprotiline increases GluA1 and GluA2/A3 subunit expression in the hippocampus [214]. The TCA imipramine enhances the synaptic expression of GluA1 and S845-GluA1 without changing the total levels of GluA1 in non-stressed rats or mice [215]. Consistently, bath application of the TCA imipramine, or the SSRI fluoxetine or citalopram, potentiates field excitatory postsynaptic potentials at TA-CA1 synapses in non-stressed rat hippocampal brain slices [216].

Similar effects have also been observed in stressed rodent models. Chronic fluoxetine treatment rescues the chronic unpredictable stress (CUS)-induced reduction of GluA1 gene (*Gria1*) expression in the hippocampal CA1 and DG regions, and restores GluA1 protein expression and AMPAR-mediated synaptic transmission in the hippocampal CA1 region [217]. Additionally, it partially rescues the AIS-induced impairment in PFC LTP [188, 191]. Chronic treatment with the SSRI fluvoxamine prevents the CMS-induced facilitation of LTD in the hippocampus [208]. Furthermore, chronic treatment with desipramine and the SSRI escitalopram prevents the CUS-induced deficit in extradimensional set-shifting [218].

Collectively, these lines of evidence suggest that regulation of AMPAR trafficking and synaptic plasticity certainly represents a viable downstream target for the conventional antidepressants. These drugs may either first target the monoamine systems and subsequently modulate AMPAR signaling, or directly target AMPAR signaling.

#### NMDAR antagonists

Considerable interest in the glutamatergic system has arisen following the empirical finding that a single dose of ketamine, originally a non-competitive NMDAR antagonist used primarily as an anesthetic, induces rapid (within 2 h), pronounced, and relatively long-lasting (7 days) antidepressant effects, even in patients with treatment-resistant depression [219, 220]. Notably, accumulating evidence indicates that AMPARs are key downstream targets of ketamine [167, 168, 173]. Acute ketamine administration elevates the protein level of BDNF, as well as surface levels of GluA1 and GluA2, and consequently increases AMPAR-mediated synaptic transmission in the CA3 and CA1 hippocampal regions of non-stressed rats or mice [221–223]. Similar effects have been observed in animal models of depression, where a single dose of ketamine completely reverses CUS-induced decreases in the protein levels of synaptic GluA1, PSD-95, and synapsin I, as well as spine density and frequency and amplitude of AMPAR-mediated EPSC in the PFC in a mTOR-dependent manner [201, 224]. Ketamine also ameliorates the social defeat stress-induced decreases in BDNF, GluA1, and PSD-95 in the PFC, CA3, and DG regions of the hippocampus in mice [203]. Ketamine-induced antidepressant behavioral responses are blocked in BDNF knock-out (KO) mice, suggesting a critical role of BDNF in mediating its effects [221]. The synaptic-potentiation and antidepressant effects of ketamine are reversed by DNQX or NBQX, antagonists of AMPARs and kainate receptors regardless of subunit composition [223, 225, 226], but not by NASPM, a channel blocker of GluA2-lacking AMPARs [223]. Moreover, the synaptic-potentiation and antidepressant-like effects of ketamine in the forced swim test or the novelty-suppressed feeding paradigm are abolished in GluA2 KO mice, suggesting that the GluA2 subunit of AMPARs plays a key role in mediating the antidepressant effects of ketamine [223]. Consistently, (2R,6R)-HNK, a major metabolite of ketamine, exerts antidepressant actions as well as potentiation of AMPAR-mediated currents and upregulation of AMPARs in hippocampal synapses, which are reversed by the AMPAR antagonist NBQX [227].

Similar to AD and HD, extrasynaptic GluN2B-containing NMDARs may play a major role in mediating the effects of stress [228]. Selective GluN2B antagonists and non-selective NMDAR antagonists, as well as allosteric modulators of NMDA channels such as GLYX-13, produce antidepressant responses in rodent models and in humans [201, 224, 225, 229]. The channel blocker memantine reverses LTP deficits in the PFC and improves CUS-induced impairment in prefrontal cortical synaptic plasticity and reversal learning, although it may impair spatial memory [206, 230, 231]. NMDAR channel blocker MK-801 (dizocilpine), and Ro25-6981, a GluN2B selective antagonist, exert antidepressant-like effects, possibly by elevating BDNF levels [221].

#### AMPAkines

AMPAR positive allosteric modulators, or AMPAkines, which potentiate AMPAR function by binding at the ligand-binding domain dimer-interface on the AMPAR complex, have also shown antidepressant effects in animal models as well as in patients. Preclinically, AMPAkines such as LY451646, LY392098, Tulrampator, piracetam, aniracetam, CX516 and CX691 exhibited antidepressant-like effects in animals models [174, 232–236]. As expected, the antidepressant effects of the AMPAkine LY392098 in the forced swim test were blocked by the non-competitive AMPAR antagonist LY300168 [233]. Interestingly, AMPAkines also induce BDNF expression [237] which is associated with antidepressant action of almost all classes of antidepressants. BDNF expression is increased in the hippocampus by different classes of antidepressants [238]. Furthermore, deletion of BDNF in the hippocampus attenuates the antidepressant behavioral responses [239–241] and intraventricular or intrahippocampal infusion of BDNF is sufficient to induce rapid, sustained anti-depressant-like effects [242, 243].

The fact that a range of mechanistically different antidepressants impact AMPAR trafficking and function supports the notion that dysregulation in AMPAR trafficking may play a central role in the pathogenesis and treatment of stress and depression.

### Dysregulation of AMPAR diffusional trapping in rodent models of stress and depression

The first evidence for a role of AMPAR diffusion in stress and depression arose from in vitro studies showing that the stress hormone corticosterone significantly impacts the diffusion of AMPAR in cultured hippocampal neurons [178]. Acute treatment with corticosterone for 5–10 min rapidly increases GluA2-AMPAR lateral diffusion via the mineralocorticoid receptor, facilitating their synaptic recruitment in response to a chemical LTP stimulus. Over several hours, corticosterone slowly increases surface GluA2-AMPAR diffusion and synaptic content via the glucocorticoid receptor, occluding further synaptic AMPAR potentiation [18, 178, 244, 245]. These findings suggest that dysregulation of AMPAR diffusional trapping may underlie impairments in synaptic plasticity and cognition observed in animal models of stress and depression. The therapeutic potential of targeting AMPAR diffusional trapping in depression has been demonstrated in experiments using the atypical antidepressant Tianeptine [18].

Tianeptine (S 1574, [3-chloro-6-methyl-5,5-dioxo-6,11-dihydro-(c,f)-dibenzo-(1,2-thiazepine)-11-yl) amino]-7 heptanoic acid, sodium salt) is a clinically used atypical antidepressant under the brand name Stablon or Coaxil. Structurally similar to tricyclic antidepressants, tianeptine is a serotonin reuptake enhancer that decreases the extracellular levels of serotonin [170, 246]. We previously demonstrated that tianeptine rapidly reduces AMPAR lateral diffusion under basal conditions and in corticosterone-treated hippocampal neurons [18]. It also reverses corticosterone- and AIS-induced impairments in hippocampal and PFC LTP [18, 182, 188, 190, 191] and prevents stress-induced memory impairment [247, 248]. Mechanistically, tianeptine promotes the interaction between stargazin and PSD-95 in hippocampal neurons via CaMKII phosphorylation of stargazin. The CaMKII inhibitor KN-93 or the expression of stargazin ΔC, which lacks the last four C-terminal amino acids corresponding to the PDZ binding site of PSD-95, prevents the tianeptine-induced surface AMPAR immobilization [18]. In addition to its role in the diffusional trapping of AMPARs, tianeptine rapidly augments the phosphorylation of the GluA1 subunit at S831 and S845 [190, 249, 250], which are involved in the surface expression and synaptic retention of AMPARs. Consistent with these findings, tianeptine potentiates AMPAR-mediated synaptic transmission by activating CaMKII and PKA via the MAPK pathways p38, MEK1/2, and JNK [250]. Notably, the antidepressant effects of tianeptine in forced swim test are blocked by AMPAR antagonist NBQX [251], further corroborating that the ability to modify AMPAR-mediated synaptic plasticity is a crucial feature of clinically effective antidepressants [170].

Taken together, the discovery that tianeptine promotes the diffusional trapping of AMPAR supports the concept that dysregulation of AMPAR diffusional trapping may underlie deficits in synaptic plasticity and cognition in models of stress and depression.

## Conclusions

The convergence of HD, AD, and depression on the dysregulation of AMPAR diffusional trapping highlights a unified pathological mechanism likely contributing to early-onset psychiatric disturbances and cognitive impairments in these conditions. Identifying this mechanistic node may open a new avenue for early therapeutic intervention, which could be particularly advantageous for complex conditions like neurodegenerative and neuropsychiatric disorders, where the exact pathogenesis remains largely unknown.

Targeting convergent mechanisms in disease treatment is often more efficient and associated with fewer side effects than targeting individual pathways because it addresses a common downstream process affecting multiple disease pathways simultaneously. This approach simplifies therapeutic intervention and development, as a single treatment can modulate a central mechanism impacting various pathways, leading to broader therapeutic benefits. Additionally, focusing on a convergent mechanism reduces the risk of off-target effects and toxicity, as it is more likely to influence specific aspects of disease pathology without disrupting overall cellular balance.

As proof of principle, we found that, in addition to its beneficial effects on models of stress and depression, the atypical antidepressant tianeptine rescues synaptic plasticity and cognitive deficits in a number of complementary HD animal models by promoting the diffusion trapping of AMPARs in a BDNF-TrkB-dependent manner [64]. These experiments suggest that, beyond its known antidepressant effect, tianeptine may be repurposed to provide cognitive benefits in HD and AD. One of the main advantages of targeting the diffusional trapping of AMPARs—the final step of the AMPAR cellular journey to the synapses—is that it is likely associated with minimal side effects. Given that AMPARs mediate the majority of fast synaptic transmission in the brain, efforts to directly regulate channel function (e.g., with ampakines) have been associated with intolerable toxicity levels [167]. Similarly, although strategies targeting extrasynaptic NMDAR activity with low-dose memantine have shown benefits in several neurodegeneration models, including AD and HD [106], clinical studies using NMDAR channel blockers for neurological conditions have been disappointing, largely due to the essential physiological role of NMDARs [252].

We suggest that specifically targeting the surface redistribution of AMPARs already present at the plasma membrane via diffusional trapping of AMPARs—without impacting the total number of surface AMPARs—may effectively restore cognitive function in these disorders without the aforementioned side effects. Ideally, therapeutics should promote the diffusional trapping of AMPAR by directly interacting with the receptor itself—without affecting its channel properties—or with components of its diffusional trapping machinery, such as PSD-95. A recent exciting study reported the design and characterization of a “synaptic organizer”—a peptide that bridges presynaptic neurexins to postsynaptic AMPARs [253], presumably stabilizing synaptic AMPAR via diffusional trapping [254]. Moreover, a recent study developed a 21-amino-acid peptide that interferes with the interaction between TARP and the N-lobe of the activity-regulated cytoskeleton-associated protein (Arc/Arg3.1), which in turn strengthens the interaction between PSD-95 and TARP, enhancing the diffusional trapping of AMPARs. Remarkably, a 7-day infusion of this peptide effectively prevented the forgetting of fear memory induced by social isolation [255]. Supporting the idea that stabilizing synaptic AMPAR could be beneficial across various neurodegenerative and neuropsychiatric disorders, this multivalent AMPAR binder restores synaptic and cognitive function in AD models, as well as motor coordination and locomotion in mouse models for cerebellar ataxia and spinal cord injuries [253]. Similarly, we previously demonstrated that artificially immobilizing AMPARs using an antibody-crosslinking approach rescues synaptic loss in an in-vitro AD model [63]. These studies underscore the potential therapeutic value of directly and specifically stabilizing synaptic AMPARs via their diffusional trapping.

Moreover, promoting AMPAR exocytosis could increase the number of surface AMPARs, potentially enhancing AMPAR diffusional trapping without altering their total number or channel properties. Since AMPARs are exocytosed to extrasynaptic sites, this strategy would expand the pool of mobile extrasynaptic AMPARs available for synaptic recruitment through diffusional trapping [118]. This aligns with numerous studies showing that activation of β-adrenergic receptors—either through noradrenaline or pharmacological agents like isoproterenol—not only increases the extrasynaptic AMPAR pool but also enhances LTP and memory [256–259]. Similarly, targeting signaling pathways that promote AMPAR exocytosis, such as PKA, ERK, and PI3K/Akt pathways, may offer therapeutic benefits in neurodegenerative disorders, including AD and HD [102, 260–262].

An important open question in the field is whether brain disorders differentially affect AMPARs based on their subunit composition. For instance, we have demonstrated that the diffusional trapping and synaptic anchoring of endogenous GluA2-containing AMPARs—likely comprising GluA1/GluA2 and GluA2/GluA3 heteromers—are disrupted in AD models [63]. In contrast, oAβ has been shown to promote the synaptic recruitment, rather than the elimination, of GluA1 homomeric calcium-permeable AMPARs, contributing to synaptic dysfunction [263, 264]. Furthermore, Aβ-induced dendritic spine loss has been found to require Ca^2+^ influx through calcium-permeable AMPARs [265]. Interestingly, the endocytic adaptor protein CALM (clathrin assembly lymphoid myeloid leukemia protein) selectively targets ubiquitinated GluA1-homomeric calcium-permeable AMPARs for endocytosis via a clathrin-independent mechanism [266]. These findings emphasize the complex interplay between AMPAR subunit composition and the pathological mechanisms underlying brain disorders. A deeper understanding of how these processes are selectively regulated could pave the way for novel therapeutic approaches to restore synaptic function.

Another key question in the field is the potential influence of genetic mutations or common variations within the human population on the diffusional trapping of AMPARs and their role in increasing susceptibility to neurological disorders. For example, it would be interesting to investigate whether the single-nucleotide polymorphism (SNP) in the *BDNF* gene (Val66Met), which is known to negatively impact learning and memory and be associated with stress-related disorders and AD [267–269], also affects the diffusional trapping of AMPARs. Similarly, it remains to be determined whether genetic variations in AMPARs or components of the diffusional trapping machinery—such as stargazin, PSD-95, and CaMKII—contribute to disease susceptibility. Notably, in this context, recent studies have shown that the V143L stargazin mutation, linked to intellectual disability, increases the surface mobility of stargazin. This is evidenced by enhanced mean square displacement and diffusion rates, as well as decreased synaptic residence time, likely resulting from impaired interactions with AMPA receptors and reduced phosphorylation [270].

It is noteworthy that neither complete destabilization nor complete immobilization of AMPAR surface diffusion is beneficial. Maintaining a physiological dynamic equilibrium is key to an effective treatment. Normal lateral diffusion allows receptor exchange between synaptic and extrasynaptic compartments, reversible binding to postsynaptic anchoring slots, and accumulation at synaptic sites or endocytic zones [271]. Aberrant destabilization of AMPARs observed in HD, AD and depression models results in impairments in synaptic plasticity and cognition [63, 64]. Conversely, abnormal immobilization of AMPAR, such as crosslinking AMPARs in naïve neurons [17], also blocks synaptic plasticity and cognition by preventing the normal diffusion of extrasynaptic AMPAR to synapses during LTP.

In conclusion, we propose that AMPAR diffusional trapping is a converging point in HD, AD, and stress-related disorders like depression and may serve as a promising early therapeutic target in diseases associated with memory deficits.

## Data Availability

Not applicable.

## Abbreviations

CNS: Central nervous system
AMPARs: AMPA receptors
NMDARs: NMDA receptors
mGluRs: Metabotropic glutamate receptors
LTP: Long-term potentiation
LTD: Long-term depression
PSD: Postsynaptic density
HD: Huntington’s disease
AD: Alzheimer’s disease
mHTT: Mutant Huntingtin
polyQ: Polyglutamine repeats
HFS: High-frequency stimulation
BDNF: Brain-derived neurotrophic factor
TrkB: Tropomyosin receptor kinase B
CaMKII: Ca^2+^/calmodulin-dependent protein kinase II
PI3K: Phosphatidylinositol 3-kinase
ERK: Extracellular signal-regulated kinase
TARPs: Transmembrane AMPA receptor-associated proteins
CREB: CAMP response element binding protein
oAβ: Oligomeric forms of amyloid beta peptide
PFC: Prefrontal cortex
SSRIs: Selective serotonin reuptake inhibitors
TCAs: Tricyclic antidepressants
EPSC: Evoked excitatory postsynaptic current
AIS: Acute inescapable stress
ARTS: Acute restraint plus tailshock stress
AFSS: Acute forced swim stress
CUMS: Chronic unpredictable mild stress
